# Allopurinol Lowers Serum Urate but Does Not Reduce Oxidative Stress in CKD

**DOI:** 10.3390/antiox11071297

**Published:** 2022-06-29

**Authors:** Mingyao Sun, Nicole Hines, Diego Scerbo, Jane Buchanan, Chaorong Wu, Patrick Ten Eyck, Diana Zepeda-Orozco, Eric B. Taylor, Diana I. Jalal

**Affiliations:** 1 Department of Internal Medicine, Division of Nephrology, Carver College of Medicine, University of Iowa, Iowa City, IA 52242, USA; mingyao-sun@uiowa.edu (M.S.); nicole-hines@uiowa.edu (N.H.); 2 Department of Molecular Physiology, University of Iowa, Iowa City, IA 52242, USA; diego641990@gmail.com (D.S.); jane-buchanan@uiowa.edu (J.B.); eric-taylor@uiowa.edu (E.B.T.); 3 Institute for Clinical and Translational Science, University of Iowa, Iowa City, IA 52242, USA; chaorong-wu@uiowa.edu (C.W.); patrick-ten-eyck@uiowa.edu (P.T.E.); 4 Center for Clinical and Translational Research, Abigail Wexner Research Institute at Nationwide Children’s Hospital, Columbus, OH 43205, USA; diana.zepeda-orozco@nationwidechildrens.org; 5 Department of Pediatrics, Division of Nephrology and Hypertension, The Ohio State University College of Medicine, Columbus, OH 43210, USA; 6 Iowa City VA Medical Center, Iowa City, IA 52242, USA

**Keywords:** xanthine oxidase, urate, chronic kidney disease

## Abstract

Xanthine oxidase (XO) contributes to oxidative stress and vascular disease. Hyperuricemia and gout are common in patients with chronic kidney disease (CKD), a population at increased risk of vascular disease. We evaluated effects of allopurinol on serum XO activity and metabolome of CKD patients who had participated in a randomized double-blind clinical trial of allopurinol vs. placebo. XO activity was measured in participants’ serum. XO expression in venous endothelial cells was evaluated via immunofluorescence. Gas chromatography mass spectrometry (GC/MS) was utilized for metabolomics analysis. We found that in patients with stage 3 CKD and hyperuricemia, allopurinol lowered serum urate while increasing serum xanthine levels. Allopurinol, however, did not significantly suppress measured serum XO activity. Of note, baseline serum XO activity was low. Additionally, neither baseline serum XO activity nor XO protein expression were associated with measures of vascular dysfunction or with systemic or endothelial biomarkers of oxidative stress. Allopurinol affected several pathways, including pentose phosphate, pyrimidine, and tyrosine metabolism. Our findings suggest that circulating XO does not contribute to vascular disease in CKD patients. In addition to inhibition of XO activity, allopurinol was observed to impact other pathways; the implications of which require further study.

## 1. Introduction

Gout and hyperuricemia are associated with several cardio-metabolic risk factors, including cardiovascular and kidney disease [1]. It is estimated that >70% of those with gout have significant chronic kidney disease (CKD) [1]. Patients with CKD represent a group at markedly increased risk of morbidity and mortality due to cardiovascular disease (CVD) [2,3,4,5,6]. Increased serum urate levels in CKD have been shown to associate with increased risk of cardiovascular events and death in this group of patients [7]. Xanthine oxidase (XO), the enzyme that converts hypoxanthine to xanthine, and xanthine to uric acid, is an important source for oxygen free radical production [8,9]. In the vasculature, XO is expressed in the endothelial cells where enhanced XO activity has been shown to contribute to increased superoxide production and to reduced nitric oxide availability (i.e., endothelial dysfunction) [10]. As such, XO may play a role in the development and progression of vascular disease in patients with CKD [11,12].

Serum urate levels are known to be elevated in CKD. This is likely related, at least partially, to reduced renal clearance of uric acid in the setting of reduced glomerular filtration rate [13]. However, it is also plausible that XO activity is increased in CKD considering the high prevalence of cardio-metabolic risk factors in this patient population. Several studies have explored the potential effect of urate-lowering on CVD outcomes in patients with CKD [14,15]. These studies have utilized the XO inhibitor, allopurinol [14,15,16,17]. Of note, no study to date has evaluated the effects of allopurinol on systemic XO activity in CKD.

Considering the high prevalence of gout/hyperuricemia in CKD, the potential links between gout/hyperuricemia, kidney disease progression and CVD in CKD, and that allopurinol is the most commonly prescribed urate-lowering agent for the treatment of gout/hyperuricemia and in clinical trials [18,19], we sought to evaluate the effects of allopurinol on XO activity in patients with CKD. Specifically, we hypothesized that XO inhibition in response to allopurinol therapy would associate with improved oxidative stress in CKD. In addition, the field of mass spectrometry-based metabolomics assessment has undergone considerable technological advances in the last few decades, providing an opportunity to obtain a comprehensive understanding of the potential impact of allopurinol on the metabolome of CKD patients [20]. In this post-hoc analysis, we utilized samples from a randomized double-blind clinical trial of allopurinol therapy in patients with stage three CKD [21].

## 2. Methods

### 2.1. Study Subjects

This is a post-hoc analysis. The rationale, design, objectives, and inclusion/exclusion criteria for ‘parent study’ have been described previously [21]. Briefly, the ‘parent study’ was a randomized double-blind placebo-controlled study of 80 patients with stage 3 CKD and asymptomatic hyperuricemia who received 300 mg of allopurinol vs. placebo for 12 weeks. We recruited adult men and women with stage 3 CKD (defined as eGFR between 30–60 mL/min/1.73^2^) [22] and asymptomatic hyperuricemia (defined as serum urate of ≥7.0 and ≥6.0 mg/dL, respectively, with no history of gout). Potential subjects were excluded from participation if they were currently taking allopurinol or another uric acid-lowering agent or if they had a history of intolerance to allopurinol. Additional exclusion criteria included: any contraindication to the use of allopurinol such as severe liver disease, a history of severe congestive heart failure, any infection within the past 2 weeks, any hospitalization within the past 3 months, use of coumadin, and a body mass index > 40 kg/m^2^. The ‘parent study’ was approved by the Colorado Multiple Institutional Review Board and all participants were required to provide written consent prior to participation in the study. All the research visits for the ‘parent study’ took place at the Clinical and Translational Research Center (CTRC) at the University of Colorado Anschutz Medical Center. For this analysis, we included all the subjects who had an adequate volume of serum samples stored at −80 °C from the baseline and end of study visits (n = 14 in each study group (allopurinol and placebo)).

### 2.2. Xanthine Oxidase Activity

XO activity of serum samples was assayed using Amplex^®^ Red Xanthine/Xanthine Oxidase Assay Kit (Thermofisher, Eugene, OR, USA) according to user’s instruction [23]. This kit measures superoxide formation in a coupled reaction to the red-fluorescent oxidation product, resofurine. Absorbance at 560 nm was measured via spectrophotometry. Samples were incubated at 37 °C in the reaction mixture for 30 min and absorbance measured at 560 nm in a standard spectrophotometer. Then, serum XO activity was determined from a simultaneously prepared standard curve. Hypoxanthine supplied in the kit was used as the standard and XO activity was measured by comparing the absorbance of samples with standards. XO activity was calculated as mU/mL of serum volume.

### 2.3. Endothelial Protein Expression

The expression of endothelial XO protein was evaluated via immunofluorescence of the venous endothelial cells that had been collected from the participants’ antecubital veins [21]. Slides were systematically scanned to identify endothelial cells (positive VE-Cadherin). Nuclear integrity was confirmed using 4′,6′-diamidino-2-phenylindole hydrochloride staining (DAPI). The immunofluorescence was quantified for the XO protein utilizing XO antibody (Abcam) in the endothelial cells expressing both VE-Cadherin and DAPI. Images were captured and then analyzed using NIS Elements BR Software (version 4.60.00, 64-bit, Nikon, Tokyo, Japan) to quantify the intensity of CY3 staining (i.e., average pixel intensity). Values for each sample were reported as ratios of endothelial cell XO protein expression to human umbilical vein endothelial cell (HUVEC) expression to account for any variation in the staining procedure. Technicians were blinded to subject identity during the staining and analysis procedures. In addition, we had previously evaluated the endothelial expression of nitrotyrosine (Abcam) [21], a footprint of oxidative injury in the endothelium [24]. The endothelial expression of nitrotyrosine (NT) was included in this analysis as an outcome.

### 2.4. Endothelium-Dependent and Independent Dilation

Endothelium-dependent dilation was evaluated via brachial artery flow-mediated dilation (BA-FMD) and endothelium-independent dilation via nitroglycerin mediation dilation (NMD). This was the primary outcome for the ‘parent study’ and was measured as described originally by Celermajer et al. [25] and subsequently by our group [26]. Briefly, BA-FMD was measured at the CTRC by a trained technician using high-resolution ultrasonography (GE Vivid 7 Dimension). Reactive hyperemia was produced by inflating a pediatric forearm cuff around the forearm to 250 mmHg for 5 min followed by rapid deflation. NMD was determined by measuring brachial artery dilation for 10 min after administration of sublingual nitroglycerin (0.4 mg). We used a commercially available software package (Vascular Analysis Tools 5.8.1, Medical Imaging Applications, LLC, Coralville, Iowa, USA) to concurrently acquire electrocardiogram (ECG)-gated brachial artery diameters during baseline, FMD, and NMD conditions. Brachial artery dilation was determined as the % change from baseline. Doppler flow of the brachial artery was also measured and peak shear rate was calculated as a potential covariate. Brachial artery dilation was determined as the % change from baseline. The images were analyzed by an independent research assistant who was blinded to the study groups.

### 2.5. Carotid Intima-Media Thickness (CIMT)

Similar to BA-FMD, CIMT was measured at the CTRC by an experience technician as previously described [27]. Briefly, the left common carotid artery was imaged via GE Vivid 7 ultrasound equipped with a linear array transducer and carotid artery diameter was analyzed using image analysis software (Carotid Analyzer version 5.10.10, Medical Imaging Applications). A longitudinal segment of the cephalic portion of the carotid artery was acquired ~2 cm distal to the carotid bulb for at least 10 cardiac cycles. Carotid IMT was defined as the distance from the leading edge of the lumen-intima interface to the leading edge of the media-adventitia interface on the far wall, measured during end diastole. All image analysis was performed by a single research assistant blinded to the study randomization.

### 2.6. Metabolomics Analysis

The analysis was conducted at the Fraternal Order of Eagles Diabetes Research Center Metabolomics Core facility in the Carver College of Medicine at the University of Iowa [28]. This targeted, in-house, standard-verified protocol measures more than 100 metabolites via gas chromatography–mass spectrometry. These include tricarboxylic acid (TCA) cycle and glycolytic/gluconeogenic intermediates in addition to amino acids, sugars, neurotransmitters, and fatty acids [29]. For metabolite extraction, the plasma samples were extracted in ice-cold 1:1 methanol/acetonitrile, which contained a mixture of 9 internal standards (d4-Citric Acid, 13C5-Glutamine, 13C5-Glutamic Acid, 13C6-Lysine, 13C5-Methionine, 13C3-Serine, d4-Succinic Acid, 13C11-Tryptophan, d8-Valine; Cambridge Isotope Laboratories, Tewksbury, MA, USA) at a concentration of 1 ug/mL each. The ratio of extraction solvent to sample volume was 18:1. The samples were then incubated at −20 °C for 1 h followed by a 10-min centrifugation at maximum speed. Supernatants were transferred to fresh tubes. Pooled quality control (QC) samples were prepared by adding an equal volume of each sample to a fresh 1.5 mL microcentrifuge tube. Processing blanks were utilized by adding extraction solvent to microcentrifuge tubes. Samples, pooled QCs, and processing blanks were evaporated using a speed-vac. The resulting dried extracts were derivatized using methyoxyamine hydrochloride (MOC) and N,O-Bis(trimethylsilyl)trifluoroacetamide (TMS) (both purchased from Sigma, St. Louis, MO, USA). Briefly, dried extracts were reconstituted in 30 μL of 11.4 mg/mL MOC in anhydrous pyridine (VWR), vortexed for 10 min, and heated for 1 h at 60 °C. Next, 20 μL TMS was added to each sample, and samples were vortexed for 1 min before heating for 30 min at 60 °C. The derivatized samples, blanks, and pooled QCs were then immediately analyzed using GC-MS.

GC chromatographic separation was conducted on a Thermo Trace 1300 GC with a TraceGold TG-5SilMS column (0.25 µm film thickness; 0.25 mm ID; 30 m length). The injection volume of 1 μL was used for all samples, blanks, and QCs. The GC was operated in split mode with the following settings: 20:1 split ratio; split flow: 24 μL/min, purge flow: 5 mL/min, Carrier mode: Constant Flow, Carrier flow rate: 1.2 mL/min). The GC inlet temperature was 250 °C. The GC oven temperature gradient was as follows: 80 °C for 3 min, ramped at 20 °C/minute to a maximum temperature of 280 °C, which was held for 8 min. The injection syringe was washed 3 times with pyridine between each sample. Metabolites were detected using a Thermo ISQ single quadrupole mass spectrometer. The data were acquired from 3.90 to 21.00 min in EI mode (70eV) by single ion monitoring (SIM). Metabolite profiling data were analyzed using Tracefinder 4.1 utilizing standard verified peaks and retention times.

We used TraceFinder 4.1 to identify metabolites in extracted samples, blank, and QCs. We do this by comparing sample metabolite peaks against an in-house library of standards. The standard library was prepared by processing and analyzing authentic standards via the method described above. We created a database of retention times and three fragment ions for each metabolite standard: a target peak/ion and two confirming peaks/ions. When running biological samples, we identify metabolites that not only match with the known retention times of the authentic standard, but also with its target and confirming peaks. Tracefinder was also used for GC-MS peak integration to obtain peak areas for each metabolite. After TraceFinder analysis, we correct for instrument drift over time using local regression analysis as described by Li et al. [30]. We use the pooled QC samples, which were run in duplicate at the beginning and end of the GC-MS run for this purpose. The data are then normalized to an internal standard (d4-Succinic Acid) to control for extraction, derivatization, and/or loading effects. Levels of the given metabolites were compared ratiometrically and are presented as fold change for each metabolite at the end of study visit normalized to the metabolite at the baseline visit with the baseline values normalized to 1.

### 2.7. Statistical Analysis

Baseline characteristics by study treatment group are presented as mean ± SD for continuous variables with categorical variables shown as %. Pearson’s chi-square test was used to evaluate the relationship between two categorical variables. Mann-Whitney test was used to access the difference of outcome variables between the two treatment groups. The vast majority of the metabolites were not normally distributed. Thus, we utilized the Wilcoxon Sign Rank test to evaluate differences in the metabolites between the baseline and end of study visits within each group. We then evaluated if the change in metabolites (from baseline to end of study) differed between the allopurinol and placebo groups via the Wilcoxon Rank Sum test. Next, we evaluated the potential correlation between the baseline levels of the metabolites and measures of vascular function and oxidative stress including BA-FMD, NMD, CIMT, serum oxidized low-density lipoprotein (oxLDL), and endothelial NT expression. Similarly, we evaluated the correlation between the baseline metabolite levels and measures of kidney function including Chronic Kidney Disease Epidemiology Collaboration (CKD-EPI) eGFR [31] and urinary albumin/creatinine ratio (ACR). SAS (version 9.4, Cary, N.C.) was used to conduct these analyses. To better characterize the pathways reflected by the metabolites that were significantly altered by allopurinol, we created a correlation network diagram in MetaboAnalyst 4.0. Metabolite Set Enrichment Analysis and Metabolomic Pathway Analysis are web-based tools that incorporated into MetaboAnalyst platform. We use those tools to perform metabolite enrichment and pathway analyses, respectively [32]. Color intensity (white to red) reflects a larger number of metabolites and higher statistical significance. Metabolites were included if they met the *p* < 0.05 threshold for the in-between group significance for the change in allopurinol vs. placebo. The color and size of each circle was based on *p*-value and pathway impact value, respectively. Significance was defined as *p* value < 0.05 for all analyses.

## 3. Results

### 3.1. Clinical Characteristics of the Participants

Twenty-eight subjects had residual samples at the baseline and end of study visits and were included in the analysis. No clinically significant differences were noted between those included in the analysis (n = 28) vs. the subjects that were not included (without residual samples). These data are shown in Supplemental Table S1. Baseline characteristics of the included subjects are presented in Table 1 according to treatment group. No significant differences were found between the placebo and allopurinol groups in demographics. Systolic and diastolic blood pressure, hemoglobin A_1C_, eGFR, ACR, and history of DM or cardiovascular disease did not differ between the groups. We, furthermore, observed no significant different in baseline BA-FMD, NMD, CIMT, oxLDL, or endothelial NT. Notably, serum urate levels, serum XO activity, and XO protein expression were similar between the allopurinol and placebo groups at baseline.

### 3.2. Correlation of Baseline XO Activity and Expression with Makers of Vascular Function and Oxidative Stress

First, we evaluated whether baseline serum XO activity correlated with baseline measures of vascular or kidney function. We found no significant relation between serum XO activity and BA-FMD, NMD, CIMT, oxLDL, or endothelial NT. These data are shown in Table 2. Similarly, we found no significant relation between the baseline endothelial expression of XO and baseline BA-FMD, NMD, CIMT, oxLDL, or endothelial NT. Of note, serum XO activity did not correlate with serum urate. Rather, when evaluating the potential relation between serum urate levels and vascular and kidney outcomes, we found a significant inverse relation of baseline serum urate levels with baseline CKD-EPI GFR (Spearman correlation coefficient = −0.58, *p* < 0.0001).

### 3.3. The Effects of Allopurinol on Serum Urate and XO Activity

**Figure 1:**
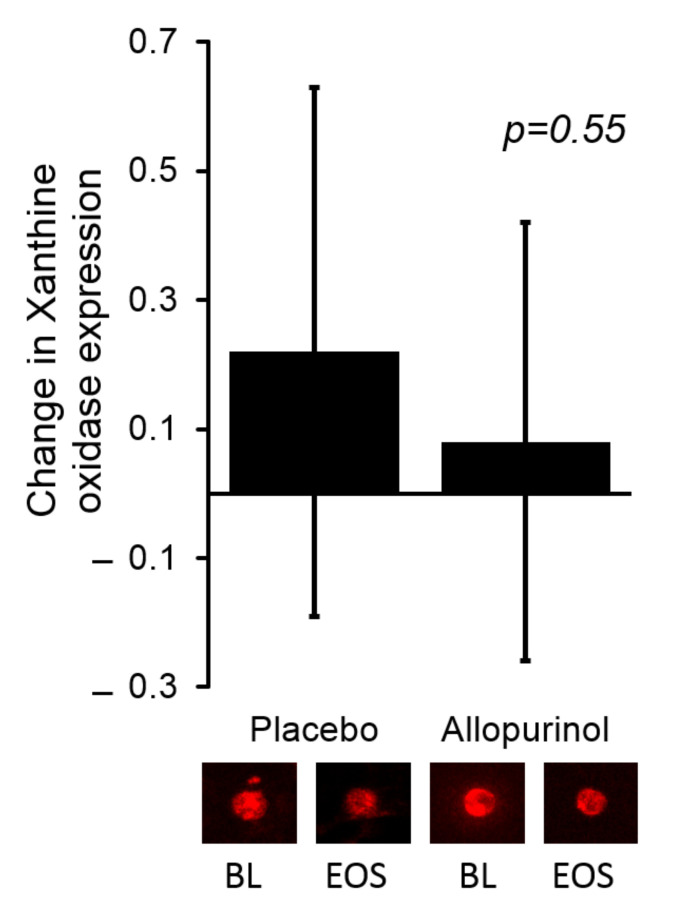
There was no significant change in the expression of endothelial XO when comparing allopurinol to placebo. The bars in the graph represent the change from baseline for each study group. Representative images of the endothelial expression of XO are shown below the bar graph for the baseline (BL) and end of study (EOS) visits for the corresponding study group. Values for each sample were reported as arbitrary units and represent ratios of endothelial cell protein expression to human umbilical vein endothelial cell (HUVEC) expression.

As shown in Table 3, allopurinol effectively lowered serum urate levels by −3.60 (−4.24, −2.96) mg/dL as compared with a change of 0.26 (−0.38, 0.89) mg/dL in the placebo group (*p* < 0.0001). Allopurinol use was associated with a significant increase in serum xanthine 7.54 (5.74, 9.90) compared to no change in the placebo group 1.02 (0.77, 1.33); in between group difference *p* < 0.0001. Of interest, we found no significant change in the serum activity of XO; the absolute change of serum XO activity was −0.14 (−0.34, 0.064) and −0.04 (−0.39, 0.09) for the allopurinol and placebo groups, respectively (*p* value = 0.70). We observed no significant change in the expression of XO protein in the endothelium of the participating individuals in either group. These data are shown in Figure 1.

### 3.4. Correlation of Change in Serum XO Activity with Makers of Vascular and Kidney Function and Markers of Oxidative Stress

We subsequently evaluated whether change in serum XO activity over the 12-week study period correlated with any of the markers of vascular function or oxidative stress. Consistent with the findings of the cross-sectional analysis, we found no significant relation between the change in serum XO activity and change in BA-FMD, NMD, CIMT, oxLDL, or endothelial NT.

### 3.5. The Effects of Allopurinol on the Metabolome in Patients with CKD

**Figure 2:**
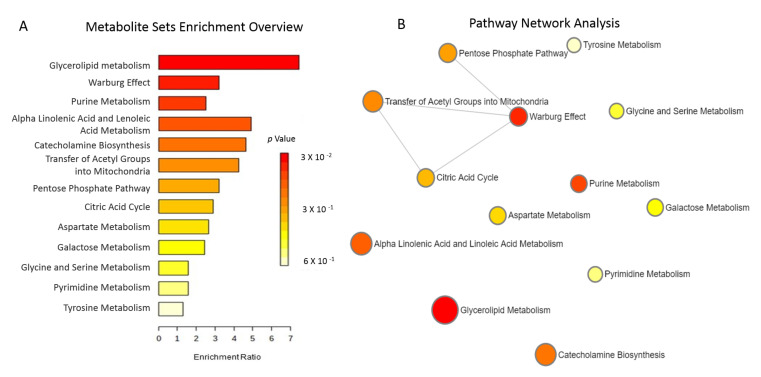
(**A**). Metabolites Sets Enrichment. Over-representation analysis including the metabolites most significantly altered by allopurinol is shown. This does not account for the direction of the change in the metabolites (increased vs. decreased). Bar colors reflect the significance (*p* value) for the specific pathway. The length of each bar indicates fold enrichment of each metabolic pathway. (**B**). Metabolic Pathway Analysis highlights the significant pathways affected by allopurinol compared to placebo. We found limited overlap between the pathways affected by allopurinol. The matched pathways are shown as circles. The *Y*-axis represents the −log of *p*-values from pathway enrichment. The *X*-axis represents pathway impact value calculated from pathway topology analysis. The color and diameter of each circle is based on *p*-value and pathway impact value, respectively.

We identified 11 metabolites that changed significantly in the group treated with allopurinol vs. placebo (Table 4). The intensities of two metabolites (xanthine and orotate) increased by approximately six and twelve fold, respectively, after twelve weeks of allopurinol treatment without notable change in the placebo group. Ribose-5-phosphate (R5P), dihydroxyphenylalanine, and N-Acetyl Tyrosine were reduced by approximately 40%, 30%, and 60% with allopurinol but did not change in placebo group. The metabolites most impacted by allopurinol vs. placebo and their corresponding pathways included: xanthine/purine metabolism, R5P/the pentose phosphate pathway, orotate/pyrimidine metabolism, and dihydrophenylalanine and N-acetyl tyrosine/tyrosine metabolism. To better characterize the pathways reflected by the metabolites that were significantly altered by allopurinol vs. placebo, a correlation network diagram was created in MetaboAnalyst. Limited overlap was observed amongst the affected pathways; the most notable being glycerolipid, purine metabolism and the Warburg effect. These data are shown in Figure 2.

## 4. Discussion

We report, for the first time, the comprehensive effects of the most-commonly prescribed XO inhibitor, allopurinol, on the metabolome of patients with stage three CKD. Importantly, we measured circulating XO activity at baseline and in response to allopurinol therapy in patients with CKD. Our analysis indicates that allopurinol lowers serum urate concentration and increases serum xanthine but does not suppress circulating XO activity (as measured in the serum). XO activity is a major source of reactive oxidative species (ROS) [33] and XO inhibition is associated with reduced ROS [34]. However, here were unable to identify a significant change in of the biomarkers of vascular inflammation or oxidative stress with allopurinol [21]. We, furthermore, were unable to identify any association between serum XO activity or endothelial XO expression and biomarkers of vascular inflammation or oxidative stress. Collectively, our data suggest that circulating XO does not contribute to vascular dysfunction or oxidative stress in patients with CKD.

Allopurinol was discovered as a result of the drug discovery program at Burroughs Wellcome, an effort that yielded several impactful drugs, including allopurinol [35]. In 1966, allopurinol was approved by the Food and Drug Administration for the treatment of gout. To this day, allopurinol is a mainstay in the treatment of gout and hyperuricemia [36]. Over the last two decades, several investigators have produced data to suggest that allopurinol may treat several cardiovascular diseases common in CKD patients, including coronary artery disease and congestive heart failure (CHF) [36]. The benefits of allopurinol have been attributed, mostly, to the improvement in endothelial function observed with the inhibition of XO [37]. XO is believed to be one of the main contributors to oxidative stress in the vascular endothelium [33]. While XO is expressed in the endothelial and smooth muscle cells of human vessels [38], a number of reports suggest that endothelial XO is derived from the circulation and that circulating XO is sufficient (with adequate substrate) to produce severe endothelial cell injury [33,39]. While several studies have shown XO inhibition with allopurinol improves endothelial function and reduces oxidative stress in patients with CHF [40,41], our group was unable to reproduce similar findings in patients with CKD and hyperuricemia [21]. Our results in this analysis may explain these discrepant findings.

First, we observed contrasting findings regarding allopurinol’s effect on XO. Specifically, allopurinol did lead to decreased serum urate and increased serum xanthine levels, which are known effects on purine metabolism. However, allopurinol did not lead to decreased XO activity as measured in the serum samples. That allopurinol would decrease serum urate and increase serum xanthine is not surprising considering that XO is abundantly expressed in the liver [42] and that allopurinol is metabolized quickly in the liver (within 1–2 h) to oxypurinol, that also inhibits XO [43]. Thus, these data are consistent with allopurinol inhibiting XO activity in the liver. Still, oxypurinol is eliminated almost entirely unchanged in the urine and, as such, accumulates in patients with reduced kidney function [43]. Based on this, we expected allopurinol treatment to result in a robust XO- inhibitory effect on measured circulating (serum) XO when compared to placebo. Contrary to our hypothesis, we observed no significant difference in serum XO activity with allopurinol vs. placebo. These findings may be explained by the notably low baseline serum XO activity in our subjects. Of note, while XO activity is reportedly detectable in the circulation of humans [44,45], it would appear that the expression of XO in the serum is extremely low vs. that of the liver or the intestine, as Sarnesto et al. were unable to detect the XO protein in human serum [42]. In addition to the seemingly low amounts of XO protein in the circulation, increased serum urate in CKD may contribute to reduced XO activity [46,47]. In CKD, serum urate accumulates due to reduced clearance of urate by the kidneys. This accumulation of uric acid in the serum of CKD patients would inhibit XO activity. Considering that the parent study required hyperuricemia as an inclusion criterion, it is not surprising that serum XO activity was low in our patients at baseline. Our data suggest that circulating XO does not contribute significantly to oxidative stress nor increased serum urate levels in CKD patients. Rather that the increased serum urate levels are largely related to reduced renal clearance. This conclusion is further supported by the lack of an association between baseline serum XO activity or endothelial XO expression with biomarkers of oxidative stress. These findings may explain why recently conducted clinical trials have shown no benefit of urate-lowering via allopurinol to slow CKD progression in patients with CKD, despite the large number of observational studies linking hyperuricemia to kidney disease [16,17].

**Figure 3:**
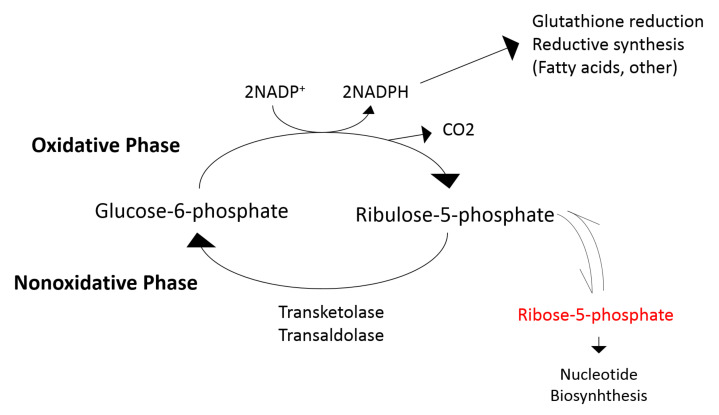
Illustration of the pentose phosphate pathway (PPP). Allopurinol-mediated inhibition of PPP reduces ribose-5-phosphate availability for purine synthesis, which likely contributes to the urate-lowering effect of the drug. Allopurinol, additionally, inhibits the oxidative branch of the PPP and may result in reduced availability NADPH.

Second, our metabolomics analysis suggests that the effects of allopurinol extend to other pathways beyond XO. Importantly, our data are consistent with allopurinol leading to decreased pentose phosphate pathway (PPP). PPP pathway (summarized in Figure 3) is a critical source of nicotinamide adenine dinucleotide phosphate (NADPH) [48], necessary for ROS-neutralizing [49]. We found that ribose-5-phosphate (R5P), which is produced by the PPP, was significantly decreased, which is consistent with PPP inhibition. The PPP, depicted in Figure 3, is also an important pathway to provide precursors for nucleotide and amino acid biosynthesis [48]. Allopurinol-induced decreases in PPP activity may contribute further to the urate-lowering effect of the drug by reducing R5P availability for purine synthesis and potentially other effects in tissues that were not observed by this study [50]. Additionally, our data are consistent with allopurinol impacting the oxidative branch of the PPP. The oxidative branch of PPP converts G6P to R5P while reducing nicotinamide adenine dinucleotide phosphate (NADP+) to NADPH [48]. NADPH is critical to protect against reactive oxygen species (ROS)-mediated damage and for the synthesis of antioxidants such as glutathione [49]. Allopurinol-mediated inhibition of the oxidative branch could decrease NADPH availability and thus antioxidant capacity. Whether the inhibitory effect of allopurinol on the PPP offsets the potential antioxidant benefit of XO inhibition requires further study.

A few additional observations warrant mention. The effects of allopurinol and its downstream metabolite, oxypurinol, on pyrimidine synthesis include inhibition of orotodine monophosphate decarboxylase (OMD) [51]. The inhibition of OMD results in the accumulation of orotidine monophosphate and its precursor orotic acid ultimately leading to orotidinuria [52]. Allopurinol-induced orotidinuria is significantly exaggerated in individuals with ornithine carbamoyltransferase deficiency and has been utilized as a diagnostic test to identify female carriers of this genetic deficiency [52]. This effect of allopurinol is likely independent of XO inhibitory effect and appears to be specific to allopurinol (a purine analogue XO inhibitor) as it is reportedly not observed in non-purine analogue XO inhibitors [53]. It is furthermore hypothesized that allopurinol may impede re-composition of high-energy phosphates via the purine salvage pathway [53]. Whether allopurinol-mediated inhibition of pyrimidine synthesis translates to clinically significant sequela in patients with CKD remains unclear.

In addition to the above, we observed a significant reduction in N-acetyl tyrosine and dihydrophenylalanine, both precursors of tyrosine [54]. Of note, it is known that CKD patients suffer from impaired hydroxylation and removal of phenylalanine, reduced synthesis of tyrosine, and the accumulation of metabolites of phenylalanine and tyrosine [54]. Our data suggest that allopurinol treatment further contributes to tyrosine deficiency in CKD patients. These findings are important, as tyrosine is a semi-essential amino acid critical in the synthesis of catecholamines including dopamine. Dopamine deficiency in the brain is a critical contributor to Parkinson’s disease [55]. Of note, allopurinol has been shown to lower serum and striatal (brain) urate in addition to striatal dopamine in animal models of Parkinson’s disease [56]. As such, the decrease in N-acetyl tyrosine and dihydrophenylalanine is likely a result of urate lowering rather than a direct effect of allopurinol or XO inhibition [57].

Lastly, we observed a significant effect of allopurinol on the glycerolipid pathway with reduced serum levels of glycerate and glycerol. Glycerol is released into the blood from adipose tissues through adipolysis and is a key component of the glycerolipid/free fatty acid cycling and critical in thermoregulation [58]. In our review of the literature, we were unable to identify other studies with a similar observation. While the exact mechanism behind this observation is unclear, fever and flushing of the skin are known potential adverse events of the drug [59]. Based on our findings, an intriguing possibility is that that allopurinol may affect thermoregulation via glycerolipid metabolism.

This work has several limitations. First it is a post-hoc analysis with a subset of participants from the parent study. Second, the parent study only recruited subjects with CKD, thus, we are unable to evaluate if serum XO activity is truly reduced in CKD vs. non-CKD subjects. Considering that our findings are limited to patients with CKD, our results are not generalizable to individuals without CKD. Nevertheless, the study has several strengths including that the parent study was a randomized placebo-controlled study, we were able to evaluate circulating and endothelial biomarkers of oxidative stress, and we offer a detailed metabolomics analysis.

## 5. Conclusions

We provide the first comprehensive evaluation of the effects of allopurinol on circulating XO activity and the metabolome of patients with CKD. Our data indicate that allopurinol, at the commonly utilized dose of 300 mg per day, lowers serum urate levels significantly in CKD patients, but (contrary to common belief), allopurinol does not appear to result in significant antioxidant effects in this patient population. This may be explained by the low activity of circulating XO in this population. These data support the use of allopurinol to lower serum urate levels in patients with an indication for such therapy (e.g., gout) but do not support the use of allopurinol to reduce ROS-mediated injury in CKD.

## Figures and Tables

**Table 1:** Subjects Baseline Characteristics.

Characteristics	Allopurinol (n = 14)	Placebo (n = 14)	*p* Value
Age (years)	61 ± 13	59 ± 8	0.27
Male sex (n (%))	10 (71)	11 (79)	>0.99
Race (n (%))			
Caucasian	9 (64)	12 (86)	0.38
African American	2 (14)	1 (7)	
Other	3 (22)	1 (7)	
Baseline diabetes	8 (57)	9 (64)	0.70
Baseline cardiovascular disease	1 (7)	1 (7)	>0.99
Systolic BP (mmHg)	126 ± 15	127 ± 15	0.96
Diastolic BP (mmHg)	76 ± 11	74 ± 10	0.5
BMI (kg/m^2^)	32.0 ± 4.2	35.1 ± 5.0	0.17
Hemoglobin A_1C_ (%)	6.6 ± 1.8	6.3 ± 1.3	0.81
Creatinine (mg/dL)	1.80 ± 0.4	1.75 ± 0.4	0.91
CKD- EPI eGFR (mL/min/1.73 m^2^)	39.9 ± 10.8	42.4 ± 11.3	0.58
ACR (mg/g)	160 ± 213	379 ± 583	0.89
BA- FMD (% change)	4.1 ± 5.4	6.2 ± 6.3	0.35
NMD (% change)	19.7 ± 8.4	16.3 ± 9.9	0.23
CIMT (mm)	0.75 ± 0.18	0.78 ± 0.19	0.93
OxLDL	43.2 ± 10.4	52.5 ± 17.9	0.08
Endothelial NT *	0.79 ± 0.14	0.87 ± 0.21	0.52
Serum urate (mg/dL)	8.4 ± 1.5	8.4 ± 1.3	0.86
Serum XO activity mU/mL	0.80 ± 0.51	0.78 ± 0.30	0.37
Endothelial XO *	1.00	1.00	>0.99

BP: blood pressure; BMI: body mass index; CKD-EPI eGFR: Chronic Kidney Disease Epidemiology Collaboration estimated glomerular filtration rate; ACR: urinary albumin/creatinine ratio; BA-FMD: brachial artery flow-mediated dilation; NMD: nitroglycerin-medicated dilation; CIMT: carotid intima-media thickness; OxLDL: oxidized low-density lipoprotein; NT: nitrotyrosine; baseline cardiovascular disease was defined as myocardial infarction, stroke, or congestive heart failure. * Values for each sample were reported as arbitrary units and represent ratios of endothelial cell protein expression to human umbilical vein endothelial cell (HUVEC) expression in order to account for any variation in the staining procedure.

**Table 2:** Baseline correlation between serum urate, XO activity, and endothelial XO expression and measures of vascular and kidney function.

Measures of Vascular Function										
Variable	BA-FMD		NMD		CIMT		oxLDL		Endothelial NT *	
	*r*	*p* Value	*r*	*p* Value	*r*	*p* Value	*r*	*p* Value	*r*	*p* Value
Serum urate (mg/dL)	−0.25	0.10	−0.3	0.07	0.29	0.06	−0.18	0.22	0.10	0.64
Serum XO activity (mU/mL)	0.002	0.99	0.04	0.80	0.14	0.39	0.12	0.41	0.22	0.30
Endothelial XO expression *	−0.27	0.41	−0.61	0.15	−0.17	0.70	−0.21	0.54	−0.1	0.90
**Measures of Kidney Disease**										
	**CKD-EPI eGFR**						**ACR**			
	***r***				***p* Value**		***r***		***p* Value**	
Serum urate (mg/dL)	−0.58				<0.0001		0.12		0.45	
Serum XO activity (mU/mL)	−0.01				0.95		0.004		0.98	
Endothelial XO expression *	0.26				0.45		0.04		0.90	

XO: xanthine oxidase; BA-FMD: brachial artery flow-mediated dilation; NMD: nitroglycerin-medicated dilation; CIMT: carotid intima-media thickness; OxLDL: oxidized low density lipoprotein; NT: nitrotyrosine; eGFR: estimated glomerular filtration rate; ACR: urinary albumin/creatinine ratio. *: Values for each sample represent ratios of endothelial cell protein expression to human umbilical vein endothelial cell expression and are reported in arbitrary units.

**Table 3:** Allopurinol, compared to placebo, did not affect serum XO activity.

Variable	Placebo	Allopurinol	*p* Value
Serum urate (mg/dL)	0.26 (−0.38, 0.89)	−3.60 (−4.24, −2.96)	<0.0001
Serum xanthine *	1.02 (0.77, 1.33)	7.54 (5.74, 9.90)	<0.0001
Serum XO activity (mU/mL)	−0.04 (−0.39, 0.09)	−0.14 (−0.34, 0.064)	0.70

Value are expressed as absolute change from baseline (interquartile range). *: expressed ratiometrically as fold change for xanthine at the end of study visit normalized to xanthine at the baseline visit with the baseline value normalized to 1. A ratio >1 indicates a significant increase of xanthine at the end of study visit.

**Table 4:** Change in metabolites according to treatment group (allopurinol vs. placebo).

	Placebo		Allopurinol		
	Median (IQR)	*p* Value *		*p* Value *	In-Between Groups ^$^
1-Octadecanol	0.99 (0.93, 1.01)	0.15	1.02 (0.98, 1.08)	0.27	0.05
2-Hydroxybutyrate	1.05 (0.87, 1.28)	0.54	1.08 (0.82, 1.34)	0.43	0.85
2-Hydroxyglutarate	1.09 (0.87, 1.24)	0.30	0.97 (0.77, 1.11)	0.39	0.17
2-Oxoadipate	1.05 (0.84, 1.25)	0.33	1.02 (0.70, 1.21)	0.86	0.29
6-Phosphogluconate	0.92 (0.84, 1.12)	0.67	0.89 (0.82, 0.97)	0.04	0.43
Adonitol	1.05 (0.93, 1.16)	0.24	0.89 (0.88, 1.02)	0.30	0.08
Alanine	1.04 (0.93, 1.29)	0.43	1.00 (0.82, 1.17)	0.86	0.85
α-Keto β-Methylvalerate	0.98 (0.85, 1.16)	0.90	0.97 (0.84, 1.21)	0.81	0.85
α-Ketoglutarate	1.03 (0.97, 1.18)	0.36	1.03 (0.93, 1.16)	0.39	0.82
α-Ketoisocaproate	0.97 (0.88, 1.09)	0.86	1.01 (0.91, 1.07)	0.71	0.89
α-Ketoisovalerate	0.99 (0.83, 1.11)	0.71	0.95 (0.82, 1.25)	0.90	0.96
Aminoadipate	1.11 (0.93, 1.29)	0.36	1.29 (1.11, 1.57)	0.09	0.12
Arachidic acid	1.02 (0.82, 1.09)	1.00	0.92 (0.85, 1.16)	0.86	0.93
Arachidonate	0.98 (0.88, 1.04)	0.50	0.90 (0.73, 1.06)	0.08	0.43
Asparagine	1.08 (0.86, 1.11)	0.90	1.03 (0.86, 1.25)	0.58	1.00
Aspartate	0.99 (0.71, 1.28)	0.76	1.09 (0.77, 1.53)	0.46	0.58
Behenic acid	1.03 (0.79, 1.11)	0.95	0.85 (0.71, 1.04)	0.01	0.10
β-Alanine	1.02 (0.96, 1.06)	0.58	1.01 (0.96, 1.08)	0.67	1.00
β-Hydroxy β-Methylbutyric.acid	1.08 (0.89, 1.17)	0.36	1.10 (0.94, 1.29)	0.14	0.68
β-Hydroxybutyrate-3	1.10 (0.73, 1.47)	0.33	1.07 (0.66, 2.15)	0.46	0.93
Cholesterol	1.04 (0.95, 1.09)	0.43	0.94 (0.88, 1.01)	0.12	0.05
Citraconate	0.97 (0.84, 1.11)	0.46	0.98 (0.93, 1.10)	0.86	0.61
Citrate ^#^	1.05 (0.93, 1.26)	0.33	0.86 (0.74, 1.00)	0.07	0.04
Citrulline	0.92 (0.81, 1.12)	0.50	0.88 (0.81, 1.03)	0.63	0.82
Cysteine	0.95 (0.86, 1.24)	0.81	1.04 (0.90, 1.12)	0.76	0.65
Cytidine	1.07 (0.94, 1.24)	0.19	1.06 (0.62, 1.20)	0.95	0.46
Cytosine	1.06 (0.94, 1.15)	0.36	0.98 (0.72, 1.28)	0.95	0.61
Dihydroxyphenylalanine ^#^	0.99 (0.92, 1.18)	0.81	0.71 (0.58, 0.88)	0.01	<0.0001
Fructose	0.91 (0.47, 1.40)	0.50	0.99 (0.40, 2.92)	0.50	0.68
Fumarate	1.06 (0.77, 1.21)	0.95	1.02 (0.86, 1.17)	0.58	0.65
γ-aminobutyric acid	1.01 (0.73, 1.20)	0.95	1.10 (0.58, 1.88)	0.46	0.82
Glucose	0.95 (0.82, 1.17)	0.86	1.01 (0.78, 1.38)	0.71	0.85
Glucose-6-phosphate	0.77 (0.42, 1.28)	0.43	1.49 (0.60, 2.58)	0.08	0.10
Glutamate	1.00 (0.92, 1.18)	0.76	1.17 (0.74, 1.64)	0.30	0.52
Glutamine	1.08 (0.93, 1.17)	0.43	1.01 (0.87, 1.17)	0.81	0.71
Glycerate ^#^	1.12 (0.86, 1.17)	0.43	0.92 (0.76, 0.98)	0.05	0.04
Glycerol ^#^	1.16 (1.00, 1.49)	0.01	0.93 (0.74, 1.34)	0.63	0.03
Glycerol Monolaurate	0.96 (0.88, 1.04)	0.63	0.93 (0.79, 1.01)	0.17	0.49
Glycine	0.99 (0.87, 1.18)	0.95	1.04 (0.78, 1.11)	0.81	0.82
Guanosine	1.10 (0.51, 1.26)	0.95	1.47 (0.91, 2.35)	0.09	0.27
Heneicosylic acid	0.99 (0.93, 1.05)	0.76	1.01 (0.94, 1.05)	0.81	0.93
Heptadecanoic acid	1.00 (0.90, 1.22)	0.76	1.03 (0.85, 1.15)	0.95	0.78
Histidine	0.97 (0.81, 1.17)	0.95	0.94 (0.85, 1.12)	0.76	0.96
Homocysteine	1.05 (0.86, 1.34)	0.33	1.00 (0.87, 1.55)	0.58	0.89
Homoserine	1.03 (0.97, 1.18)	0.15	0.98 (0.95, 1.13)	0.86	0.27
Hypotaurine	1.02 (0.73, 1.41)	0.71	0.93 (0.85, 1.40)	0.71	0.82
Hypoxanthine	1.00 (0.66, 1.15)	0.76	1.11 (0.83, 1.37)	0.24	0.31
Inotisol	1.00 (0.89, 1.25)	0.67	0.88 (0.78, 1.16)	0.71	0.46
Isoleucine	1.01 (0.78, 1.33)	0.71	1.00 (0.89, 1.47)	0.58	0.52
Itaconic acid ^#^	0.85 (0.68, 1.14)	0.39	1.43 (0.82, 1.67)	0.07	0.04
Lactate	1.06 (0.86, 1.16)	0.50	0.92 (0.83, 1.10)	0.30	0.25
Lauric acid	1.08 (0.87, 1.18)	0.54	1.01 (0.62, 1.44)	0.90	0.68
Leucine	1.02 (0.87, 1.23)	0.67	0.98 (0.87, 1.26)	0.67	1.00
Linoleate	1.06 (1.02, 1.32)	0.09	0.92 (0.84, 1.10)	0.43	0.13
Linolenic acid ^#^	1.16 (0.82, 1.72)	0.15	0.87 (0.64, 1.05)	0.06	0.02
Lysine	1.05 (0.98, 1.12)	0.12	0.95 (0.85, 1.24)	0.71	0.52
Malate	1.00 (0.81, 1.28)	0.50	0.97 (0.88, 1.09)	0.58	0.68
Malonate	0.97 (0.91, 1.16)	0.63	1.01 (0.97, 1.07)	0.76	0.49
Mannose	1.10 (0.89, 1.33)	0.24	1.06 (0.99, 1.24)	0.24	0.82
Methionine	1.02 (0.91, 1.20)	0.54	0.96 (0.79, 1.21)	0.90	0.55
Myristic.acid	1.03 (0.83, 1.61)	0.43	1.05 (0.75, 1.34)	0.81	0.65
N-acetyl aspartate ^#^	1.03 (0.97, 1.10)	0.36	0.93 (0.90, 1.01)	0.04	0.04
N-acetyl glutamate	1.00 (0.93, 1.28)	0.76	0.91 (0.79, 1.03)	0.39	0.18
N-acetyl serine	0.95 (0.78, 1.14)	0.81	0.87 (0.78, 0.99)	0.30	0.52
N-acetyl tyrosine ^#^	0.97 (0.85, 1.18)	0.86	0.41 (0.29, 0.45)	<0.0001	<0.0001
Oleic acid	1.08 (0.91, 1.61)	0.19	0.90 (0.76, 1.37)	0.81	0.27
O-Phosphoethanolamine	0.99 (0.90, 1.15)	0.76	1.01 (0.86, 1.21)	0.63	0.71
Ornithine	1.08 (0.89, 1.27)	0.39	1.07 (0.80, 1.19)	0.81	0.89
Orotate ^#^	0.90 (0.87, 1.14)	0.81	11.82 (8.97, 18.04)	<0.0001	<0.0001
Palmitate	1.05 (0.91, 1.31)	0.33	0.97 (0.85, 1.19)	0.86	0.38
Phenylalanine	1.02 (0.86, 1.20)	0.76	0.95 (0.89, 1.20)	1.00	0.96
Phosphoenolpyruvate	0.99 (0.92, 1.08)	0.95	0.94 (0.87, 0.98)	0.01	0.12
Proline	0.95 (0.79, 1.08)	0.71	1.00 (0.69, 1.30)	0.90	0.85
Pyruvate	1.10 (0.96, 1.46)	0.14	1.01 (0.77, 1.28)	0.67	0.38
Ribose	1.01 (0.83, 1.45)	0.63	0.91 (0.66, 1.35)	0.95	0.58
Ribose-5-phosphate ^#^	0.98 (0.79, 1.28)	0.81	0.58 (0.48, 0.61)	<0.0001	<0.0001
Sedoheptulose	1.06 (0.76, 1.23)	0.76	1.00 (0.91, 1.33)	0.50	0.85
Serine	1.08 (0.83, 1.23)	0.71	0.94 (0.80, 1.19)	0.81	0.78
Serotonin	0.98 (0.85, 1.27)	0.67	1.06 (0.90, 1.14)	0.58	0.65
Stearate	0.97 (0.88, 1.13)	0.95	1.02 (0.86, 1.14)	0.90	1.00
Succinate	1.08 (0.93, 1.22)	0.43	0.97 (0.86, 1.06)	0.30	0.18
Threonine	0.97 (0.80, 1.19)	1.00	0.80 (0.71, 1.45)	0.90	0.36
Thymine	1.08 (1.05, 1.12)	0.33	1.01 (0.84, 1.06)	0.46	0.10
Tryptophan	1.03 (0.92, 1.08)	0.50	0.97 (0.86, 1.08)	0.67	0.75
Tyrosine	1.01 (0.98, 1.11)	0.67	0.93 (0.82, 1.17)	1.00	0.89
Uracil	1.02 (0.90, 1.15)	0.36	0.98 (0.92, 1.11)	0.71	0.75
Urea	1.07 (0.86, 1.21)	0.58	0.99 (0.83, 1.14)	0.76	0.46
Uridine	1.06 (0.92, 1.08)	0.58	1.05 (0.90, 1.27)	0.30	0.61
Valine	1.03 (0.89, 1.30)	0.50	0.93 (0.88, 1.16)	1.00	0.71
Xanthine ^#^	1.04 (0.70, 1.26)	1.00	6.14 (3.61, 8.44)	<0.0001	<0.0001

Values are expressed ratiometrically as fold change for each metabolite at the end of study visit normalized to the metabolite at the baseline visit with the baseline value normalized to 1. A ratio >1 indicates a significant increase with allopurinol treatment. *: Wilcoxon Sign Rank test. ^$^: Wilcoxon Rank Sum test. ^#^: In-between groups significant.

## Data Availability

Restrictions apply to the availability of these data. Data were obtained from VA Eastern Colorado Health Care System, Denver, CO and are available from the authors with the permission of the VA Eastern Colorado Health Care System via a data use agreement. This can be accomplished by contacting the research compliance office at the Rocky Mountain Regional VAMC (ADDRESS: 1700 N. Wheeling St.; Research—151, Aurora, CO 80045-5701, phone: 303-399-8020).

## Supplementary Materials

The following supporting information can be downloaded at: https://www.mdpi.com/article/10.3390/antiox11071297/s1, Table S1: Baseline characteristics of the subjects included vs. those excluded from this analysis.
