# Supplementary material for: Allopurinol Lowers Serum Urate but Does Not Reduce Oxidative Stress in CKD
Source: Antioxidants (Basel). 2022 Jun 29;11(7):1297. doi: 10.3390/antiox11071297 (PMC9312025; doi:10.3390/antiox11071297)
Supplement: Supplementary file 1 [file antioxidants-11-01297-s001.zip › antioxidants-1733041-supplementary.pdf]

Table S1. Baseline characteristics of the subjects included *vs*. those excluded from this analysis.

Characteristic	Analysis Sample (n=28)	Excluded Observations (n=52)	p-value
Age (years)	60 ± 11	59 ± 11	0.85
Male sex (n[%])	21 (75)	43 (83)	0.41
Race (n[%])			0.13
Caucasian	22 (79)	37 (71)	
African American	2 (7)	12 (23)	
Other	4 (14)	3 (6)	
Baseline diabetes (n[%])	18 (64)	30 (59)	0.63
Baseline CVD (n[%])	12 (43)	24 (47)	0.72
Systolic BP (mmHg)	128 ± 15	129 ± 16	0.92
Diastolic BP (mmHg)	76 ± 10	79 ± 9	0.19
BMI (kg/m^2)	33.5 ± 4.7	31.6 ± 5.4	0.08
Hemoglobin A_{1C} (%)	13.7 ± 1.6	13.7 ± 1.8	0.86
Creatinine (mg/dL)	1.8 ± 0.4	1.8 ± 0.4	0.35
CKD- EPI eGFR (mL/min/1.73m^2)	39.3 ± 9.3	43.4 ± 9.4	0.07
ACR (mg/g)	414 ± 570	421 ± 645	0.21
